# Supplementary material for: Development of multifunctional membranes via plasma-assisted nonsolvent induced phase separation
Source: Nat Commun. 2024 Feb 5;15:1092. doi: 10.1038/s41467-024-45414-9 (PMC10844271; doi:10.1038/s41467-024-45414-9)
Supplement: Supplementary file 1 — Supplementary Information [file 41467_2024_45414_MOESM1_ESM.pdf]

Supplementary Information

Development of Multifunctional Membranes via Plasma-Assisted Nonsolvent Induced Phase Separation

Yueh-Han Huang[1], Meng-Jiy Wang[2], Tai-Shung Chung[1,2,3*]

[1]Graduate Institute of Applied Science and Technology, National Taiwan University of Science and Technology, Taipei 106335, Taiwan

[2]Department of Chemical Engineering, National Taiwan University of Science and Technology, Taipei 106335, Taiwan

[3]Department of Materials Science and Engineering, National Taiwan University of Science and Technology, Taipei 106335, Taiwan

Corresponding author: Tai-Shung Chung

Email: chencts@mail.ntust.edu.tw

Phone: (+886)-2-2733-3141 #5118

This supplementary information file contains 18 pages.

Supplementary Discussions

Supplementary Note 1. Effects of working distance and scan speed on wettability

In addition to the scan cycle, a further investigation was conducted to assess the influences of plasma working distance and scan speed on the wettability of membranes. As depicted in Supplementary Figure 3a, when the working distance is ≤ 5 cm, plasma treatment consistently renders membranes with superhydrophobic and self-cleaning characteristics after 5-9 scan cycles. In contrast, when the working distance is extended to 10 cm, plasma treatment for 1 – 9 cycles only marginally enhances the membranes' hydrophobicity (105°-115° vs. 95° of NIPS). In addition, the P10-s1 ~ P10-s9 membranes all possess non-slippery surfaces, highlighting the importance of using a short working distance to achieve the desired membrane wettability.

The scan speed is another crucial parameter similar to the working distance (Supplementary Figure 3b). At the same distance of 1 cm and 9 scan cycles, the WCA of the modified membranes decreases from ~170° to ~140° as the y-speed increases from 50 mm/s to 250 mm/s. Moreover, water can only roll-off the membrane surface when the y-speed is ≤ 100 mm/s, suggesting that a slower scan speed is preferable to effectively prepare superhydrophobic and self-cleaning membranes. In summary, to fabricate a superhydrophobic and self-cleaning membrane via plasma treatment, parameters including the scan cycle ≥ 5, working distance ≤ 5 cm, and scan speed ≤ 100 mm/s are necessary.

Supplementary Note 2. Tensile tests

Supplementary Figure 6 illustrates the mechanical properties of both NIPS and PANIPS membranes. It is noteworthy to observe that both the maximum tensile strain and maximum tensile stress peak at the plasma scan for 3 cycles. In the meanwhile, the

Young's modulus remains at the similar level. This phenomenon can be attributed to the gradual elimination of macrovoids, which are typically identified as mechanical weak points (Fig. 2). By scanning for 5 cycles, the membrane is in the intermediate stage of transitioning from a bicontinuous structure to a nodular structure. Therefore, the Young's modulus experiences a significant decrease, while the maximum tensile stress and strain remain comparable to those of the NIPS membrane. However, once the PANIPS membranes fully adopt the non-connected nodular structure (after 7 and 9 scans), their mechanical properties undergo a drastic decline. Consequently, P1-s7 and P1-s9 membranes are unable to withstand a high pressure during LEP tests.

Supplementary Note 3. The normalized mass changes and the crystalline phase compositions in different preparation methods

The water absorption rates of membranes made from PANIPS and VIPS methods were compared to that of the SANIPS (y = 0.003x+0.6487, adopted from [1]) method, and the results were summarized in Supplementary Figure 8a. In addition, three control membranes were prepared using the NIPS, VIPS, and SANIPS methods to compare their crystalline phases with the PANIPS membrane (Supplementary Figure 8b). The NIPS membrane was prepared by immediately immersing the as-cast membrane in a water coagulant bath for 20 hr. VIPS was prepared by exposing the as-cast membrane in a humid environment for 315 s before transferring it to a coagulant bath. SANIPS was prepared by spraying compressed air at 2 bar with a working distance of 20 cm on the as-cast membrane for 315 s before immersing it in a coagulant bath. PANIPS membranes (P2.5-s9) were prepared by Ar plasma scanning at a working distance of 2.5 cm for 9 cycles. The relative humidity for all membranes were conducted at ~ 80%. The coagulant bath temperature was controlled at 25 °C during phase inversion.

Supplementary Note 4. DCMD tests for P1-s9 membranes

According to the wettability tests, it is expected that PANIPS membranes with scan cycles >5 would all exhibit superhydrophobic properties with good fouling resistance. Moreover, as depicted in Supplementary Table 3, the mean pore size consistently increases with the number of plasma scan cycles, which correlates well with the SEM images. However, due to the weak mechanical properties of P1-s5 to P1-s9, directly measuring their mean pore sizes becomes challenging. Nevertheless, an anticipated increase in pore size when prolonging the plasma scan cycle can be inferred from the trend observed in the SEM cross-sectional images. Hence, it is expected that the flux could be further enhanced by using the P1-s9 membrane.

The P1-s9 membrane was then applied to treat both a 10 wt% NaCl solution and a solution containing 1000 ppm Rose Bengal dye. As demonstrated in Supplementary Figure 10, P1-s9 maintained a stable flux with rejections above 99%, regardless of the solution type. Its fouling resistance was also verified. However, using a 10 wt% NaCl solution as the feed, the flux of the P1-s9 membrane was not higher but similar to that of the P1-s5 membrane, contradicting to our initial expectation. Considering the weak tensile strength and loosely connected nodular structure of the P1-s9 membrane, it was suspected that the membrane underwent compression and densification during the MD measurements. Consequently, the flux of P1-s9 decreased to a level akin to that of P1-s5.

Supplementary Figures

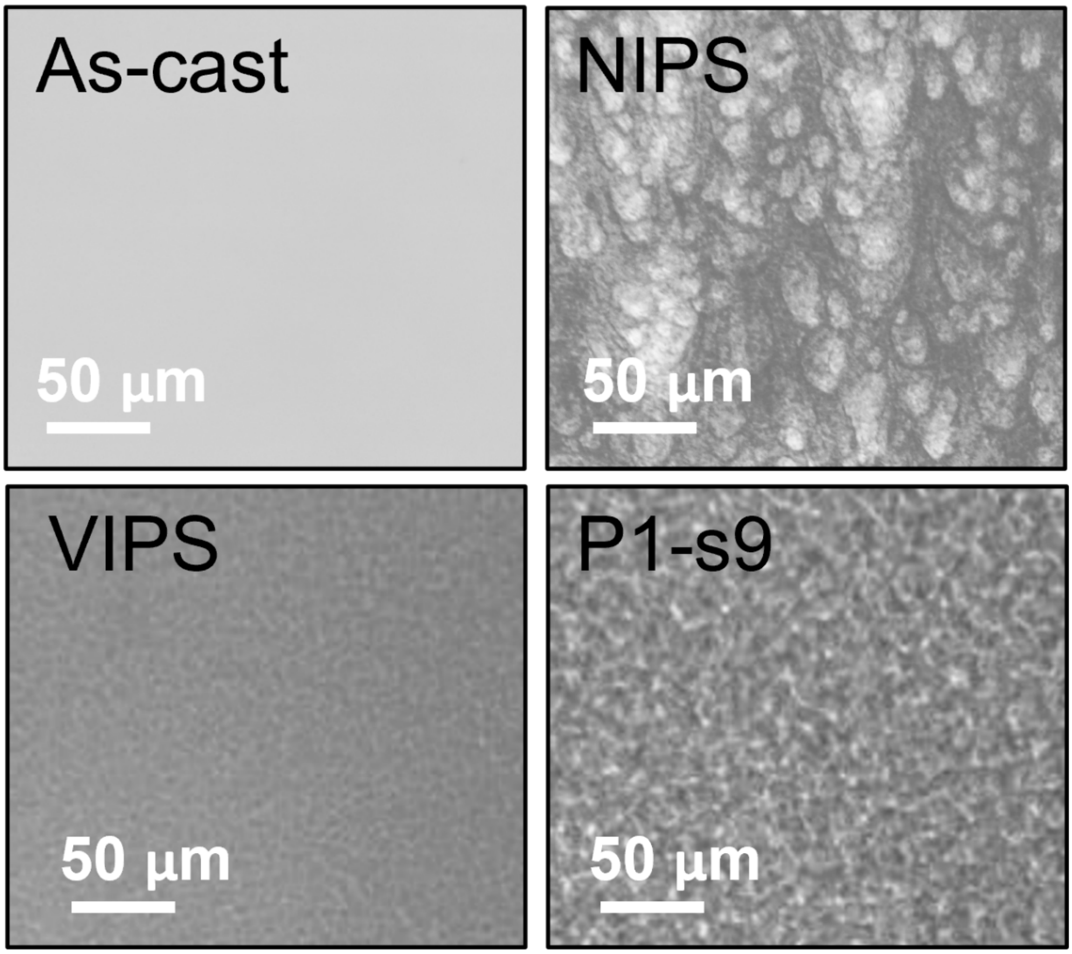

Supplementary Figure 1. The phase contrast images of the as-cast PVDF membrane and those prepared by NIPS, VIPS, and PANIPS (P1-s9) methods. The NIPS membrane was prepared by immersing the as-cast membrane in a water bath for 10 s. The VIPS membrane was prepared by exposing the membrane to humid air for 315 s (the same period for P1-s9). The relative humidity was controlled at 65-70% during the preparation.

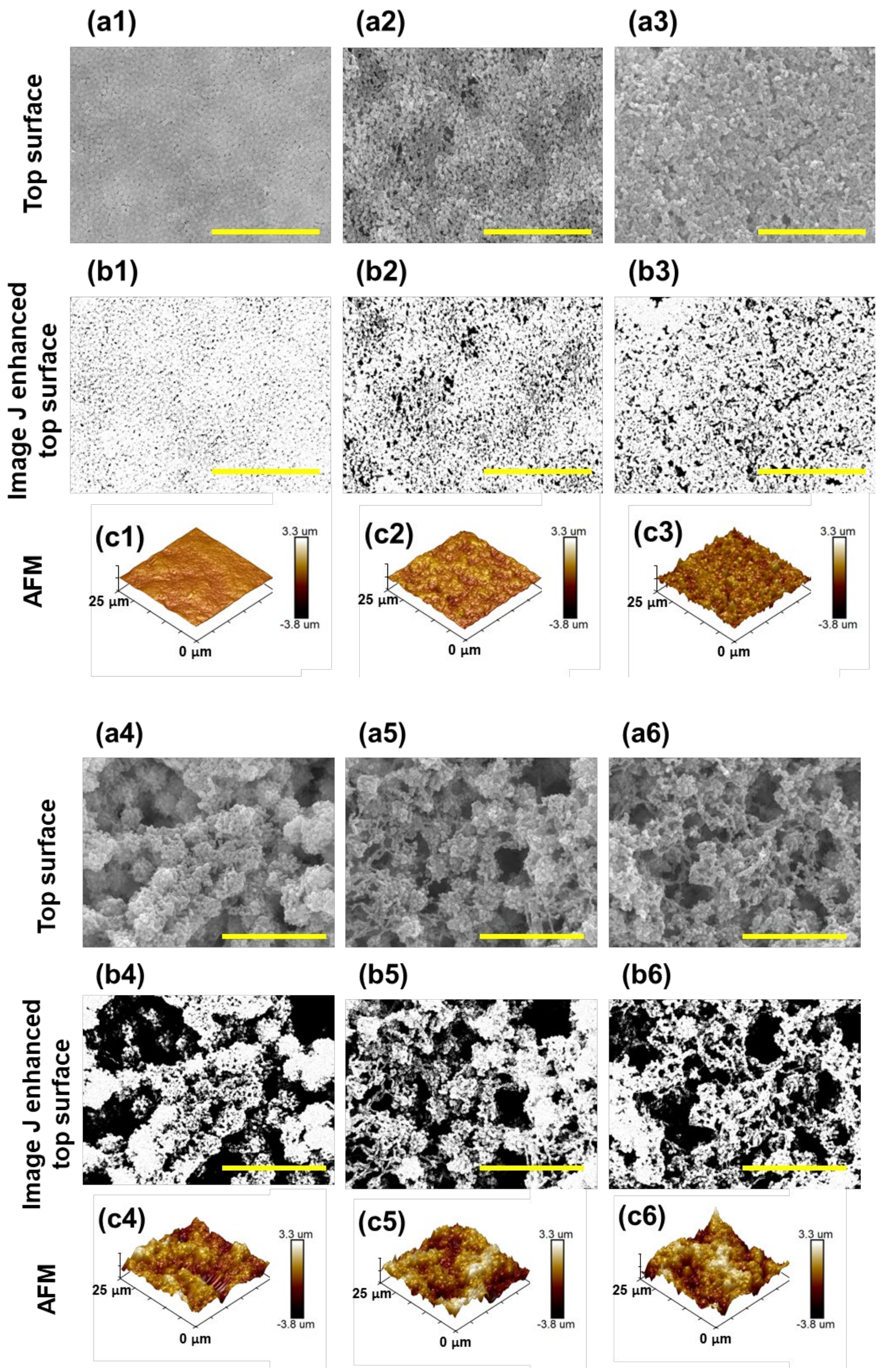

Supplementary Figure 2. Surface porosity and AFM images of the NIPS and PANIPS membranes. (a) The SEM top view images, (b) contrast enhanced Image J images, and (c) AFM images of (1) NIPS and PANISP membranes (2) P1-s1, (3) P1-s3, (4) P1-s5, (5) P1-s7, and (6) P1-s9. The images in (a) was processed by Image J software to enhance the contrast between the polymer domain and the empty porous region to get results in (b) for the calculations of surface porosity. Scale bar: 5 µm.

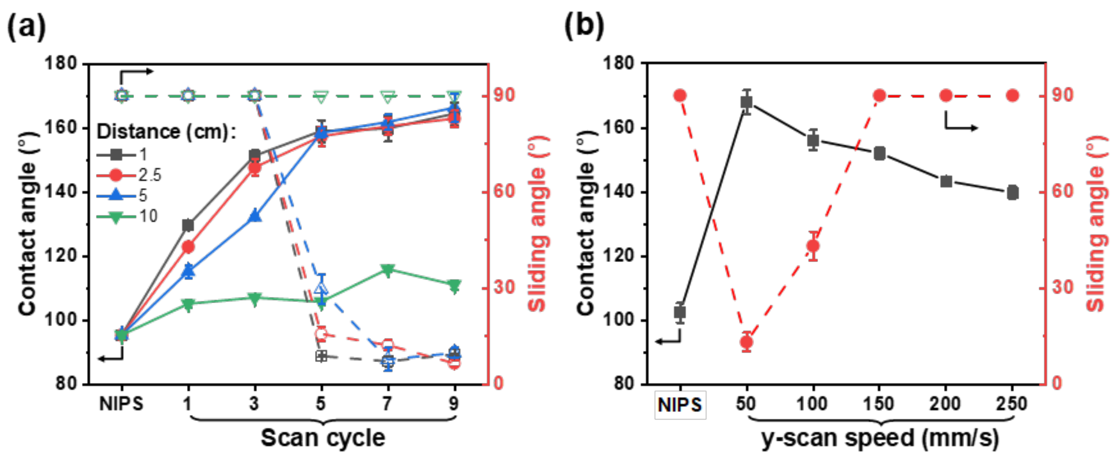

Supplementary Figure 3. Effects of (a) working distance and scan cycle, and (b) y-scan speed on water contact and sliding angle. The plasma was operated at a working distance of 1 cm and a scan cycle of 9 in (b). All data were presented as the mean of 4 replicates ± standard deviation.

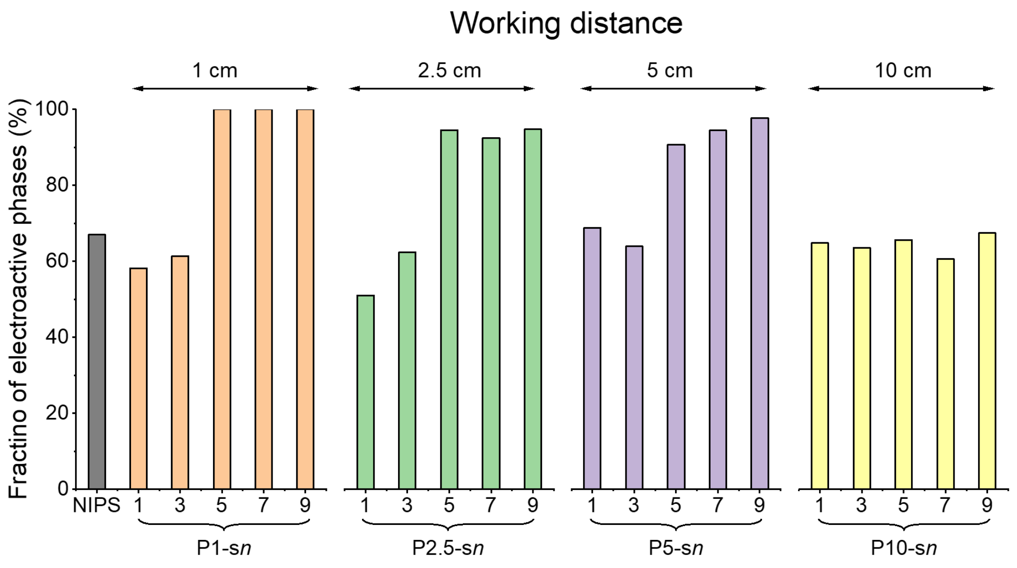

Supplementary Figure 4. The fractions of electroactive phases in NIPS and PANIPS membranes treated at various working distances (1 to 10 cm) for 1 to 9 cycles. *n* in the x-axis represents the scan cycle.

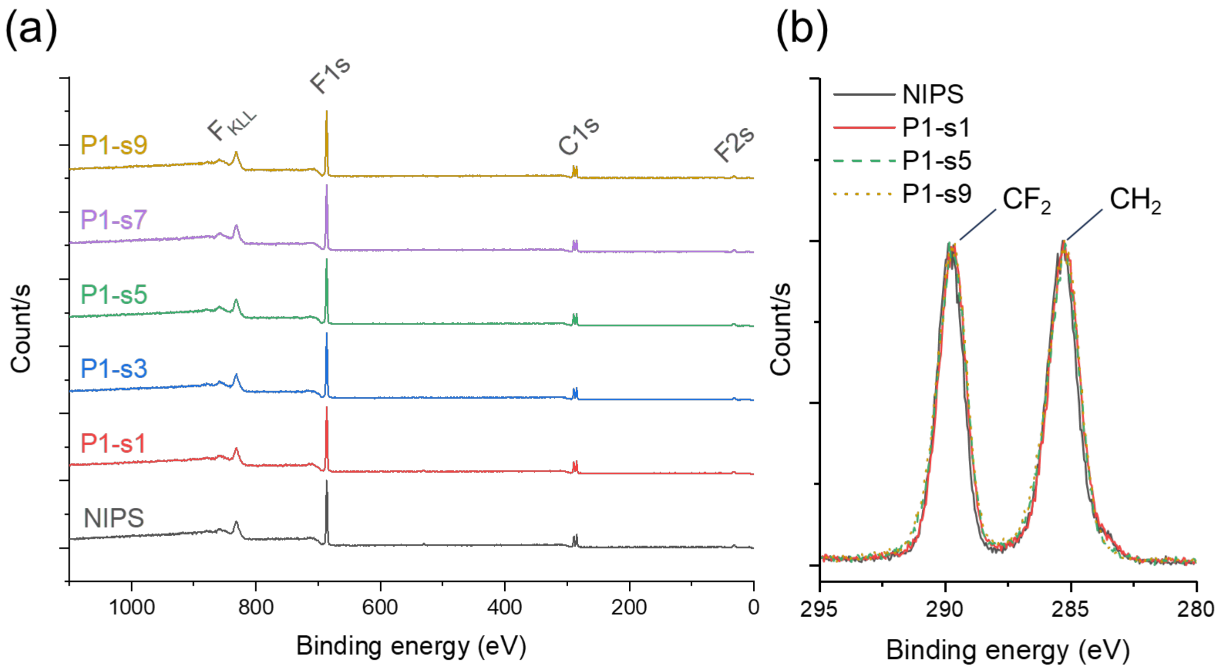

Supplementary Figure 5. Elemental compositions of the PANIPS membranes. (a) XPS survey spectra and (b) C1s narrow scan spectra of the NIPS and PANIPS membranes.

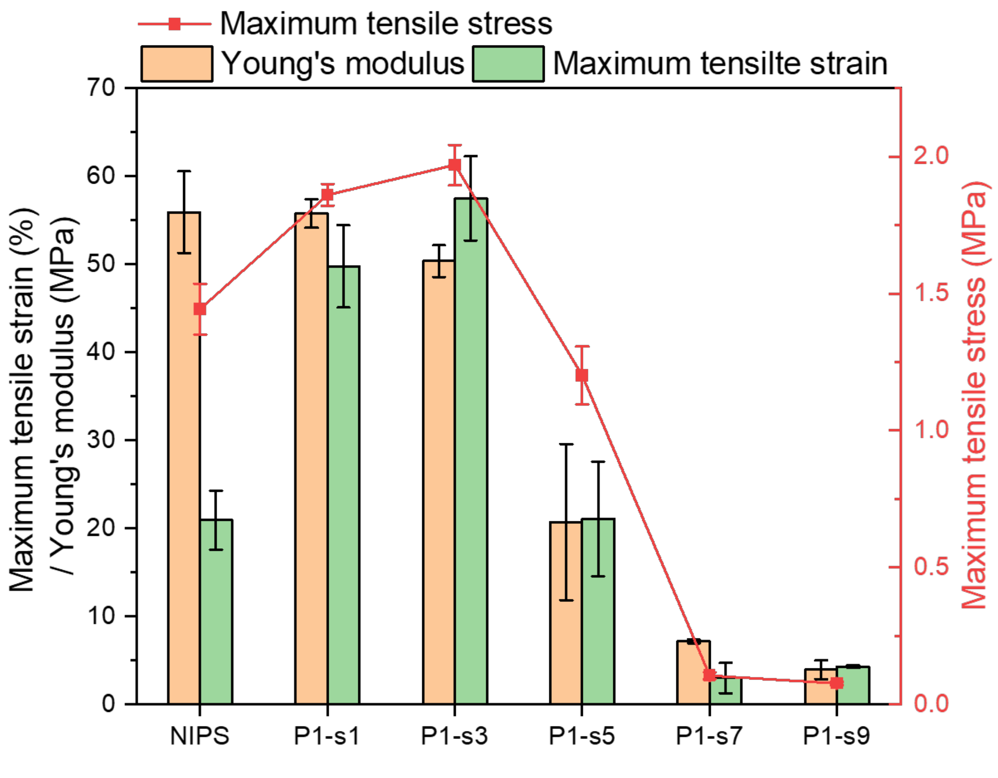

Supplementary Figure 6. Mechanical properties of the NIPS and PANIPS membranes. Data were presented as the mean of 3 replicates ± standard deviation.

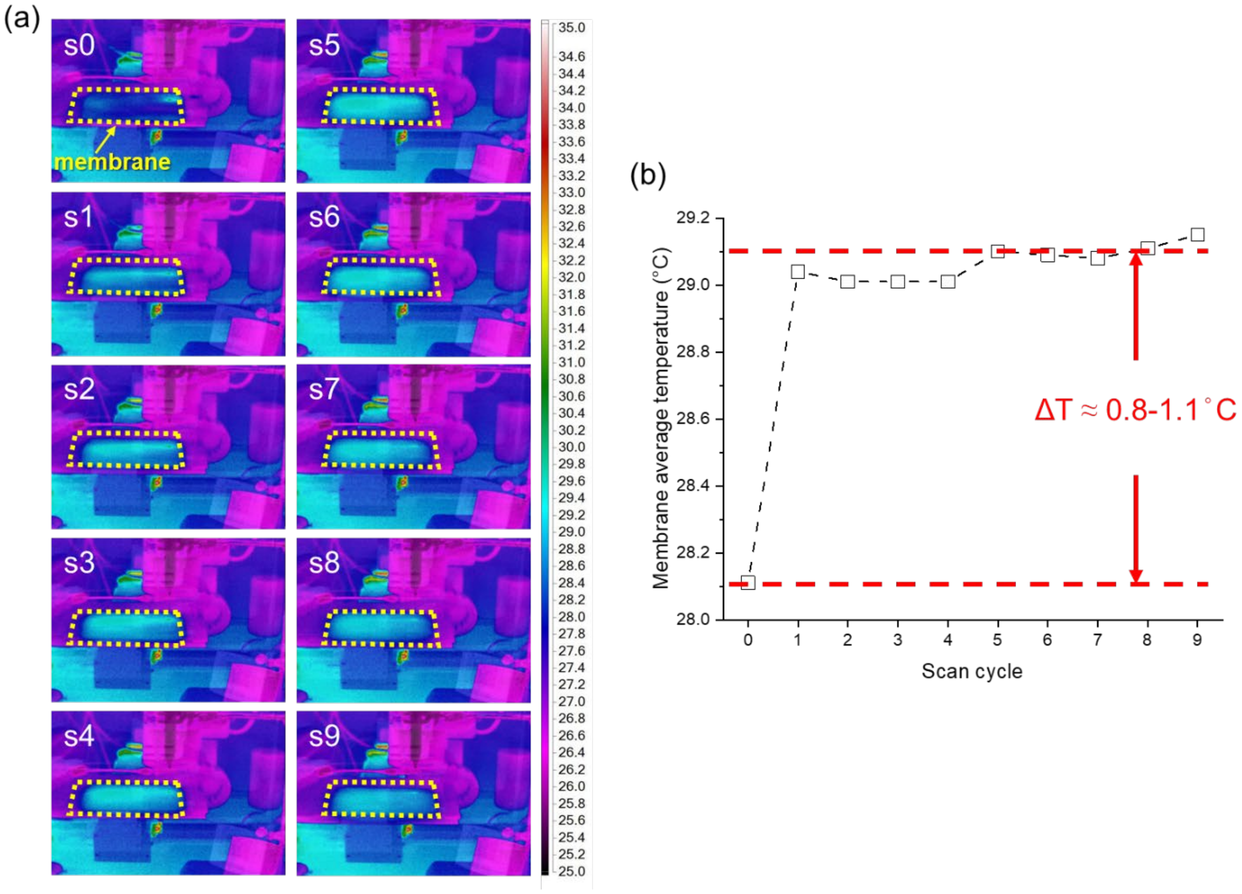

Supplementary Figure 7. Temperature profiles of the as-cast membranes during plasma treatment. (a) Infrared thermography images capturing the temperature evolution of PANIPS membrane formation across 0-9 scan cycles. The yellow line indicates the membrane area subjected to plasma scanning at a 1 cm distance. (b) The average membrane temperature derived from the corresponding images in (a).

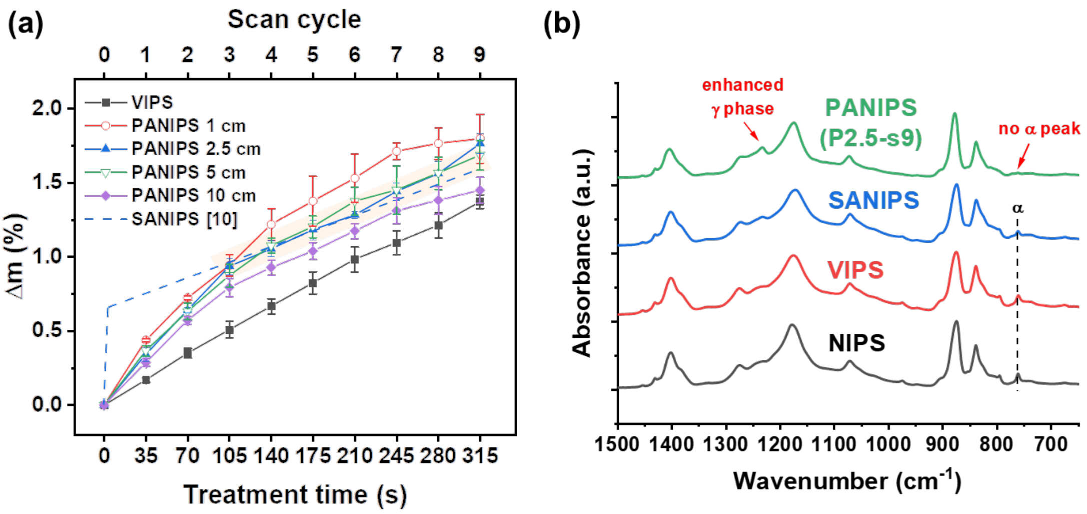

Supplementary Figure 8. Comparisons of water absorption rates and crystalline phase compositions using various preparation methods. (a) The weight-normalized mass change of the VIPS, SANIPS, and PANIPS membranes. Data were presented as the mean of 3 replicates ± standard deviation. The blue dash line in (a) represents the normalized mass change of the SANIPS method using y=0.003x+0.6487, adopted from Lu et al.[1] (b) The IR spectra of the PVDF membranes prepared by NIPS, VIPS, SANIPS, and PANIPS (P2.5-s9) methods.

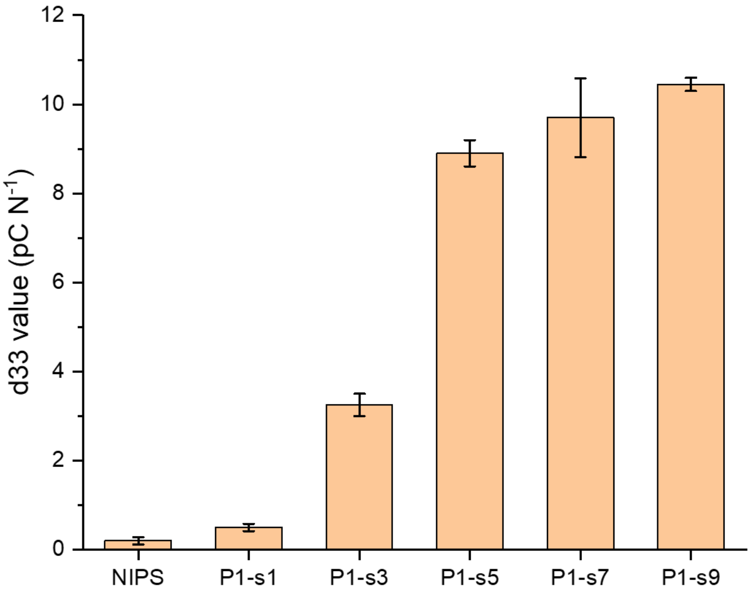

Supplementary Figure 9. Piezoelectric coefficient d33 values of the NIPS and PANIPS membranes. Data were presented as the mean of 3 replicates ± standard deviation.

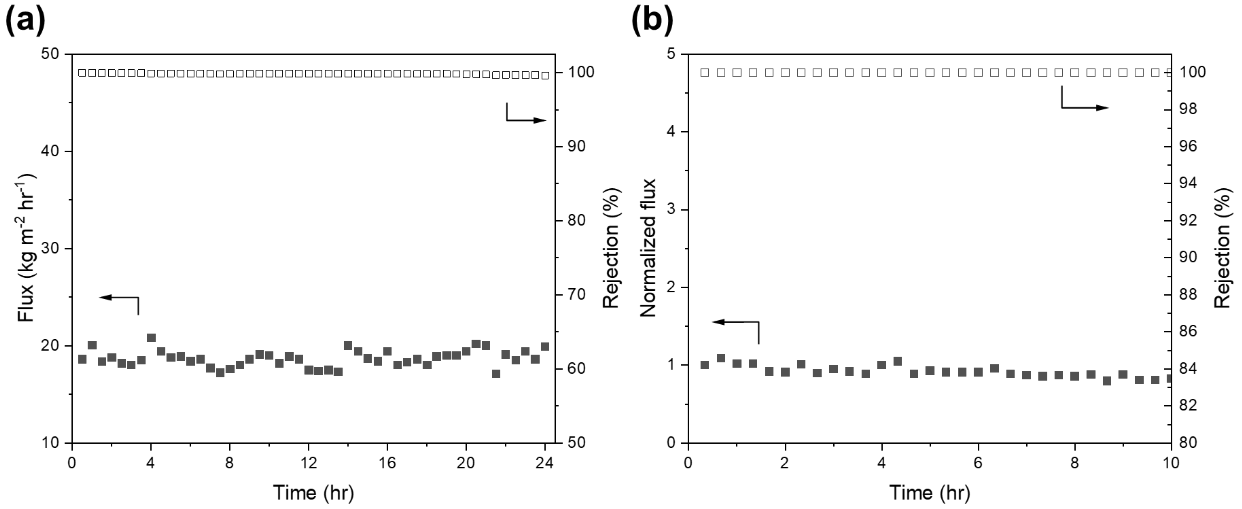

Supplementary Figure 10. DCMD tests of P1-s9 membranes with different feed solutions. (a) 10 wt% NaCl and (b) 1000 ppm Rose Bengal dye solution in 10 wt% NaCl. The feed solution was maintained at 60 °C. DI-water was used as permeate initially and controlled at 15 °C. The flux of P1-s9 was normalized by its original value of 16.2 kg m^{-2} hr^{-1}.

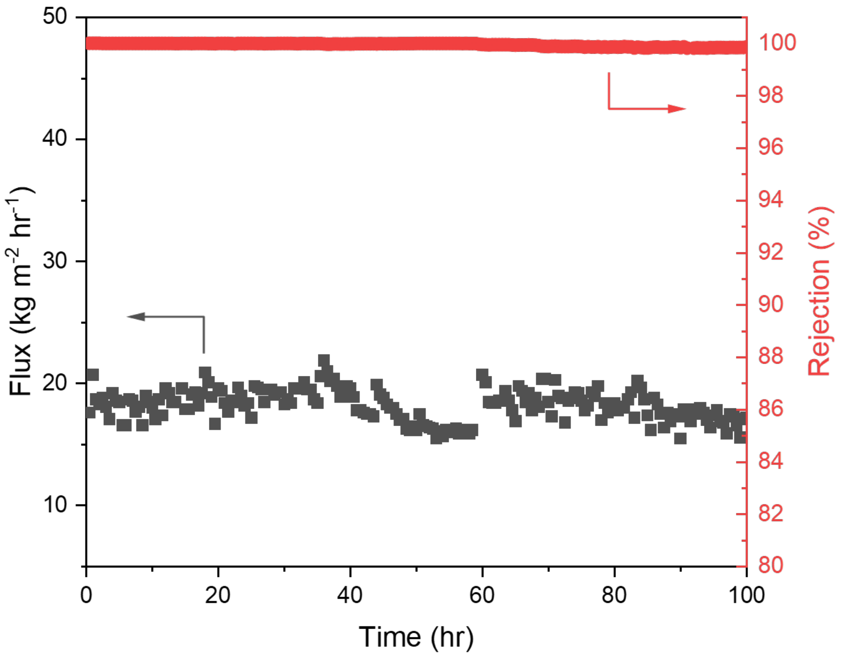

Supplementary Figure 11. Long-term DCMD performance of P1-s5 using a feed solution of 100 ppm Rose Bengal dye in 5 wt% NaCl. The feed solution was maintained at 60 °C. DI-water was used as permeate initially and controlled at 15 °C.

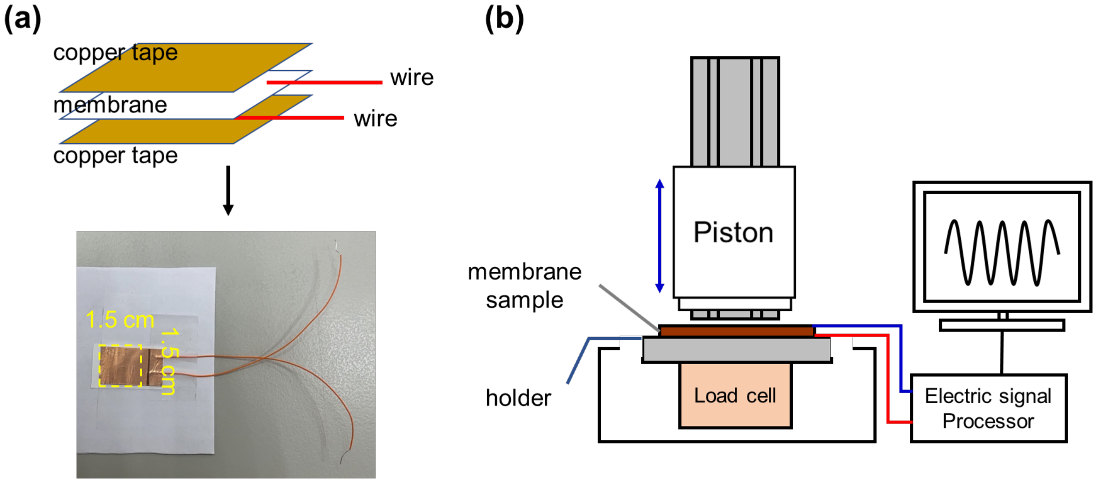

Supplementary Figure 12. Piezoelectric property tests. (a) The sample preparation by sticking copper tapes on both sides of the membrane as counter electrodes. Two wires connected to each side of the membrane were used to transport the signal to the signal processor. (b) An illustration of the reciprocating testing machine. The blue arrow next to the piston indicates the move direction. One reciprocating cycle includes: (1) The piston moves downward to apply a pressure on the membrane, measured by a load cell beneath the holder. (2) Once the force reaches the set-value, the piston will move upward to release the pressure. The procedure works at a fixed frequency of 1.82 Hz. [2, 3]

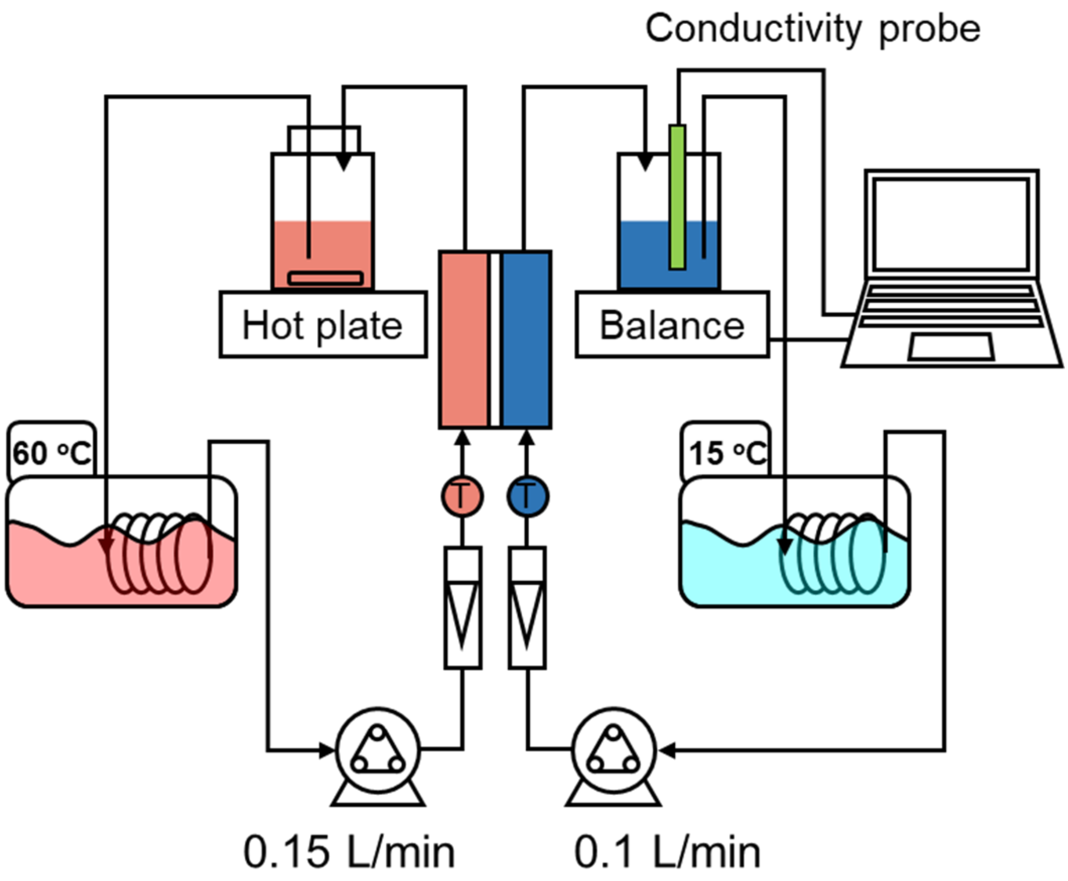

Supplementary Figure 13. Experimental setup of the DCMD system.

Supplementary Tables

Supplementary Table 1. A summary of extrinsic and intrinsic methods to fabricate highly hydrophobic to superhydrophobic PVDF membranes.

	**Membrane**	**Fabrication method**	**Modification method**	**WCA / SA**	**Ref.**
Extrinsic methods	PVDF flat sheet membrane	Commercial membrane	Pyrolysis-adhesion of ZnO nanorods, followed by PDTS modification	WCA: 152°<br>SA: N.A.	4
	PVDF electrospun nanofiber membrane	Electrospinning and heat-pressed for 2 hr	Spray-coating of CNTs/ethanol dispersion	WCA: 130° ~159.3°<br>SA: N.A.	5
	PVDF-HFP electrospun nanofiber membrane	Electrospinning	Electrospraying SiNPs during electrospinning, followed by solvent vapor welding and silanization with PDTS	WCA: 155.6°<br>SA: 7.2°	6
	PVDF flat sheet membrane	Commercial membrane	Pretreated by Ar plasma, followed by CF_4 plasma treatment at 150 W for 5-60 min	WCA: 147° ~ 162°<br>SA: N.A.	7
	PVDF hollow fiber membrane	dry-jet wet spinning	Dip-coating with Teflon® AF2400	WCA: 151°<br>SA: N.A.	8
Intrinsic methods	PVDF flat sheet membrane	NIPS using alcohols (methanol, ethanol, n-propanol, and n-butanol) as coagulants	-	WCA: 144° ~ 148°<br>SA: N.A.	9
	PVDF flat sheet membrane	NIPS using an ethanol/water mixture as a coagulant	-	WCA: 150°<br>SA: N.A.	10
	PVDF flat sheet membrane	VIPS at 24°C and 60% RH for 2 h	-	WCA: 137°<br>SA: N.A.	11
	PVDF-HFP flat sheet membrane	VIPS at 30°C and 70% RH for 20 min	-	WCA: ~144°<br>SA: N.A.	12
	PVDF flat sheet membrane	SANIPS sprayed with air, ethanol, and water at 25°C and 60% RH	-	WCA: 151.9° ~ 156.2°<br>SA: 9.6°~22.6°	1
	PVDF flat sheet membrane	SANIPS sprayed with air for 60 s at 25°C and 55% RH	-	WCA: 166°<br>SA: 20°	13
	PVDF flat sheet membrane	PANIPS using Ar microplasma	-	WCA: 164.4°<br>SA: 9.5°	This work

PVDF: poly(vinylidene difluoride); PVDF-HFP: Poly(vinylidenefluoride-co-hexafluoropropylene); PDTS: 1H, 1H, 2H, 2H-perfluorodecyltriethoxysilane; CNTs: carbon nanotubes; N.A.: not available. NIPS: non-solvent induced phase separation; VIPS: vapor-induced phase separation; SANIPS: spray-assisted nonsolvent induced phase separation

Supplementary Table 2. A summary of preparation methods and performances of piezoelectric PVDF

Membrane	Preparation steps	Crystalline phase composition	Piezoelectric performance	Piezoelectric constant, d33	Ref.
tri-layer (PVDF-BTO/n-Gr/PVDF-BTO) PNG	a layer of n-Gr sandwiched between two layers of PVDF blended with BTO, followed by poling at 100 °C by applying 15 MV/m for 1 h	only β peak was observed in XRD	output voltage: 10 V_{pp}	N.A.	[14]
PVDF–MWCNTs nanofibre mats	Electrospin using 5 wt% MWCNTs in PVDF/DMF/acetone at 18 kV	68% β phase with a total crystallinity of 38.1%	output voltage: 6 V	N.A.	[15]
Commercial PVDF film	Stretching 4 times and corona poling at 80°C for 45 min (V_N=15 kV, V_G= 2 kV)	85% β phase	output voltage (at 1 N): ~4 V_{pp}	34.3 pC/N	[16]
PVDF:Graphene membrane	PVDF and graphene mixed with the ratio 1:1.5 in NMP, cast on glass and dried at 100°C for 2 hr	Graphene induced full β phase in the membrane	output voltage: 2 V_{pp}	-11.3 pC/N	[17]
Commercial PVDF membrane	Electric poling under the electric field of 2 MV/m at 50°C for 2 hr	88.6% β phase	N.A.	5.13 pm/V	[18]
PVDF/DMAc-rGO	Electrospin using 1 wt% rGO in PVDF/DMAc/Acetone at 18 kV	69% β phase with a total crystallinity of 47.89%	output voltage: 6 ± 1.4 V	11.9 pC/N	[19]
PVDF/$BaTiO_3$ membrane	Near field electrospinning using 4 wt% $BaTiO_3$ in 15 wt% PVDF/DMSO/Acetone at 3 kV	69.7% β phase	output voltage (at 20 N): 5.44 V_{pp}	5 pC/N	[20]
PVDF/CNT foam	Solvent evaporation from a solution containing PVDF/NaCl/MWCNT with a ratio of 2/14/0.2 in 10 ml DMF	65% β phase	output voltage (at 0.02 kgf and 60% RH): 8 V	9.4 pC/N	[21]
PVDF membrane	PANIPS at 1 cm for 7 or 9 cycles	100% electroactive phase (β+γ)	output voltage (at 1 N): >10 V_{pp} for P1-s7	10.5 pC/N for P1-s9	This work

BTO: barium titanate; n-Gr: amino-treated graphene; PNG: piezoelectric nanogenerator; MWCNTs: multi-walled carbon nanotubes; V_N: needle voltage; V_G: grid voltage.

Supplementary Table 3. The mean pore sizes of the PANIPS membranes measured by the wet-up dry down method.

Membrane ID	Mean pore size (μm)
NIPS	0.104
P1-s1	0.138
P1-s3	0.187
P1-s5	N.A.
P1-s7	N.A.
P1-s9	N.A.

N.A. Not available because the membrane breaks during measurements

Supplementary References

1. Lu KJ, Zhao D, Chen Y, Chang J, Chung TS. Rheologically controlled design of nature-inspired superhydrophobic and self-cleaning membranes for clean water production. *Npj Clean Water* **3**, (2020).
2. Widakdo J*, et al.* Effects of co-solvent-induced self-assembled graphene-PVDF composite film on piezoelectric application. *Polymers* **15**, 137 (2023).
3. Kanokpaka P*, et al.* Self-powered molecular imprinted polymers-based triboelectric sensor for noninvasive monitoring lactate levels in human sweat. *Nano Energy* **100**, 107464 (2022).
4. Wang M*, et al.* ZnO nanorod array modified PVDF membrane with superhydrophobic surface for vacuum membrane distillation application. *ACS Appl Mater Interfaces* **10**, 13452-13461 (2018).
5. Yan K-K, Jiao L, Lin S, Ji X, Lu Y, Zhang L. Superhydrophobic electrospun nanofiber membrane coated by carbon nanotubes network for membrane distillation. *Desalination* **437**, 26-33 (2018).
6. Su C, Horseman T, Cao H, Christie K, Li Y, Lin S. Robust superhydrophobic membrane for membrane distillation with excellent scaling resistance. *Environ Sci Technol* **53**, 11801-11809 (2019).
7. Yang C*, et al.* CF_4 plasma-modified superhydrophobic PVDF membranes for direct contact membrane distillation. *J Membr Sci* **456**, 155-161 (2014).
8. Lu K-J, Zuo J, Chung TS. Tri-bore PVDF hollow fibers with a super-hydrophobic coating for membrane distillation. *J Membr Sci* **514**, 165-175 (2016).
9. Kuo C-Y, Lin H-N, Tsai H-A, Wang D-M, Lai J-Y. Fabrication of a high hydrophobic PVDF membrane via nonsolvent induced phase separation. *Desalination* **233**, 40-47 (2008).
10. Pagliero M, Bottino A, Comite A, Costa C. Novel hydrophobic PVDF membranes prepared by nonsolvent induced phase separation for membrane distillation. *Journal of Membrane Science* **596**, (2020).
11. Abdulla AlMarzooqi F, Roil Bilad M, Ali Arafat H. Improving liquid entry pressure of polyvinylidene fluoride (PVDF) membranes by exploiting the role of fabrication parameters in vapor-induced phase separation VIPS and non-solvent-induced phase separation (NIPS) processes. *Appl Sci* **7**, 181 (2017).
12. Chang H-Y, Venault A. Adjusting the morphology of poly (vinylidene fluoride-co-hexafluoropropylene) membranes by the VIPS process for efficient oil-rich emulsion separation. *J Membr Sci* **581**, 178-194 (2019).
13. Lu K-J, Liang CZ, Chen Y, Chung TS. Unlock the secret of air blowing in

developing high strength and superhydrophobic membranes for membrane distillation. *Desalination* **527**, (2022).

14. Yaqoob U, Uddin AI, Chung G-S. A novel tri-layer flexible piezoelectric nanogenerator based on surface-modified graphene and PVDF-BaTiO3 nanocomposites. *Appl Surf Sci* **405**, 420-426 (2017).
15. Yu H, Huang T, Lu M, Mao M, Zhang Q, Wang H. Enhanced power output of an electrospun PVDF/MWCNTs-based nanogenerator by tuning its conductivity. *Nanotechnology* **24**, 405401 (2013).
16. Mahadeva SK, Berring J, Walus K, Stoeber B. Effect of poling time and grid voltage on phase transition and piezoelectricity of poly (vinyledene fluoride) thin films using corona poling. *J Phys D: Appl Phys* **46**, 285305 (2013).
17. Huang CH*, et al.* Tailoring of a piezo-photo-thermal solar evaporator for simultaneous steam and power generation. *Adv Funct Mater* **31**, 2010422 (2021).
18. Su YP, Sim LN, Li X, Coster HG, Chong TH. Anti-fouling piezoelectric PVDF membrane: Effect of morphology on dielectric and piezoelectric properties. *J Membr Sci* **620**, 118818 (2021).
19. Sukumaran S, Szewczyk PK, Knapczyk-Korczak J, Stachewicz U. Optimizing Piezoelectric Coefficient in PVDF Fibers: Key Strategies for Energy Harvesting and Smart Textiles. *Advanced Electronic Materials*, 2300404 (2023).
20. Kong H, Jin Y, Li G, Zhang M, Du J. Design and Fabrication of a Hierarchical Structured Pressure Sensor Based on BaTiO3/PVDF Nanofibers via Near-Field Electrospinning. *Advanced Engineering Materials*, 2201660 (2023).
21. Badatya S, Bharti DK, Sathish N, Srivastava AK, Gupta MK. Humidity sustainable hydrophobic poly (vinylidene fluoride)-carbon nanotubes foam based piezoelectric nanogenerator. *ACS Appl Mater Interfaces* **13**, 27245-27254 (2021).
